# Unconventional Repertoire Profile Is Imprinted during Acute Chikungunya Infection for Natural Killer Cells Polarization toward Cytotoxicity

**DOI:** 10.1371/journal.ppat.1002268

**Published:** 2011-09-22

**Authors:** Caroline Petitdemange, Pierre Becquart, Nadia Wauquier, Vivien Béziat, Patrice Debré, Eric M. Leroy, Vincent Vieillard

**Affiliations:** 1 INSERM UMR-S 945, Immunité et Infection, Hôpital Pitié-Salpêtrière, Paris, France; 2 Université Pierre et Marie Curie, Paris, France; 3 Unité des Maladies Virales Emergentes, Centre International de Recherches Médicales de Franceville, Franceville, Gabon; 4 UMR 224 IRD/CNRS/UM1, Montpellier, France; Washington University School of Medicine, United States of America

## Abstract

Chikungunya virus (CHIKV) is a worldwide emerging pathogen. In humans it causes a syndrome characterized by high fever, polyarthritis, and in some cases lethal encephalitis. Growing evidence indicates that the innate immune response plays a role in controlling CHIKV infection. We show here that CHIKV induces major but transient modifications in NK-cell phenotype and function soon after the onset of acute infection. We report a transient clonal expansion of NK cells that coexpress CD94/NKG2C and inhibitory receptors for HLA-C1 alleles and are correlated with the viral load. Functional tests reveal cytolytic capacity driven by NK cells in the absence of exogenous signals and severely impaired IFN-γ production. Collectively these data provide insight into the role of this unique subset of NK cells in controlling CHIKV infection by subset-specific expansion in response to acute infection, followed by a contraction phase after viral clearance.

## Author Summary

Chikungunya virus (CHIKV) infection, which is responsible for devastating human illness, is rapidly becoming a global concern. The spread of this disease throughout tropical areas, where it now affects nearly 40 countries, underlines the need to improve our understanding of this infection. In 2008, CHIKV was listed as a US National Institute of Allergy and Infectious Disease (NIAID) category C priority pathogen. Natural killer (NK) cells are cytotoxic effector cells that play a vital role in the innate immune system by limiting acute infection, as previously described for several other diseases. This report describes the first phenotypic and functional analysis of NK cells soon after infection by this virus. The key element of this study was the detailed analysis of the expansion of NK cells. Coexpression of NKG2C activating receptors and HLA-C1 ligands is associated with viral load, impaired IFN-γ production, and significant cytolytic functions. We found that NK cells were able to sense CHIKV from the beginning of infection and contributed to the clearance of the infected cells through the expansion of a unique NK-cell subset.

## Introduction

Emerging and re-emerging viral infectious diseases can cause devastating illnesses in humans and are accordingly one of the principal challenges in global health care today. Among these viruses, arthropod-borne arboviruses are especially important because many of them cause fatal diseases in humans and animals [1], [2]. Chikungunya virus (CHIKV) is an *Aedes* mosquito-borne alphavirus from the *Togaviridae* family. Typically, a silent incubation period of 2–4 days usually follows infection by CHIKV, and symptoms arise afterwards. CHIKV-associated disease is an acute illness characterized by fever, skin rash, and severe incapacitating arthralgia [2]–[4]. Most clinical symptoms of CHIKV infection generally resolve within a few weeks, except for joint stiffness and pain, the hallmark of chronic CHIKV infection, which can persist for months or years. Intriguingly, CHIKV, which has historically been transmitted by *Aedes aegypti* mosquitoes, has repeatedly been associated in recent years with a new vector, *Aedes albopictus*, which has spread into tropical areas and dispersed worldwide [5]. CHIKV has been identified in nearly 40 countries and is currently present in Asia, the United States, Central Africa and Europe; the US National Institute of Allergy and Infectious Disease (NIAID) listed it as a category C priority pathogen in 2008 [2], [6], [7]. These recent outbreaks have resulted in more detailed descriptions of its clinical manifestations, including complications such as maternal-fetal transmission and fatal hemorrhagic and neurologic manifestations [8], [9].

Although much is known about this disease, the study of immune response to CHIKV infection is in its infancy. Reports in humans and in a macaque model show that virus elimination occurs very rapidly, before the host can mount IgG and T cell responses and thus suggest an effective innate immune response. Type-1 interferon (IFN) has been detected at high levels in the serum of infected individuals, its concentration correlated with viral load [10]–[14]. Natural killer (NK) cells are also an important mediator of the innate immune defense during early infectious events. NK cells mediate their antiviral effects through at least three different mechanisms: (1) the release of immunoregulatory cytokines, particularly IFN-γ, which enhance the innate immune response and help to shape the subsequent adaptive immune response, (2) the production of cytolytic granules for lysis of infected cells, and (3) the induction of target-cell death through cell surface receptors [15], [16].

The variety of signaling pathways to stimulate NK cells equips these cells with multiple detection systems for sensing and responding to infection and thus makes it more difficult for viruses to escape detection and defense. NK-cell function is controlled by the integration of signals from various activating and inhibitory cell surface receptors [17]–[19]. Its direct cytolytic activity is associated with downregulation of major histocompatibility complex (MHC) class I molecules. Different types of NK-inhibitory receptors have been identified, including the killer cell immunoglobulin (Ig)-like receptor (KIR) family, CD94/NK2A, and ILT-2. These inhibitory receptors recognize self-molecules constitutively expressed on host cells. NK cells, however, are only activated when activating receptors, including NKG2C, NKG2D, DNAM-1, and the natural cytotoxicity receptors (NKp30, NKp44 and NKp46), are triggered on NK cells at the same time that inhibitory receptors engage. NK functions are therefore finely tuned by crosstalk between the expression of activating and inhibitory receptors, and NK cells serve as important sentinels of the immune system, working as first responders and alerting the host of the presence of infectious organisms [18], [19].

Different studies have demonstrated the importance of NK cells in controlling human viral infections, such as EBV, CMV, and HSV [20]. Nonetheless, the involvement of these cells in response to alphaviruses has not been characterized either thoroughly or consistently. For example, the *in vitro* culture of Ross River virus was reported to result in enhanced rather than depressed NK cell activity [21]. More recently, Alsharifi *et al.* [22] demonstrated that NK cells without marked cytotoxic T cell involvement control the acute virulent Semliki Forest virus infection of the central nervous system in C57BL/6J mice. The frequency and activation rate of NK cells increase during acute CHIKV infection [23]. In another arboviral infection, Azeredo *et al.* [24] observed that most NK cells from dengue-infected patients display early activation markers and cell adhesion molecules during the acute phase of the disease. More recently, Hershkovitz *et al.* [25] showed that interaction of the NKp44 activating NK receptor with the flavivirus envelope protein mediates the triggering of NK cells in both West Nile and dengue viruses. Intriguingly, several flaviviruses may attenuate NK cell cytotoxicity by increasing cell surface expression of MHC class-I molecules to overcome susceptibility to NK cell mediated lysis [26], [27].

The aim of this study was to conduct a detailed phenotypic and functional analysis of NK cells during acute infection by this emerging disease, to characterize the role of NK cells during CHIKV infection. Our data, collected at a very early point post-infection, showed engagement of a clonal expansion of CD94/NKG2C^+^ NK cells that expressed receptors for HLA-C1 alleles. We describe their functional features.

## Results

### Changes in the proportion and activation status of different lymphocytic subsets from CHIKV-infected patients

**Figure 1:**
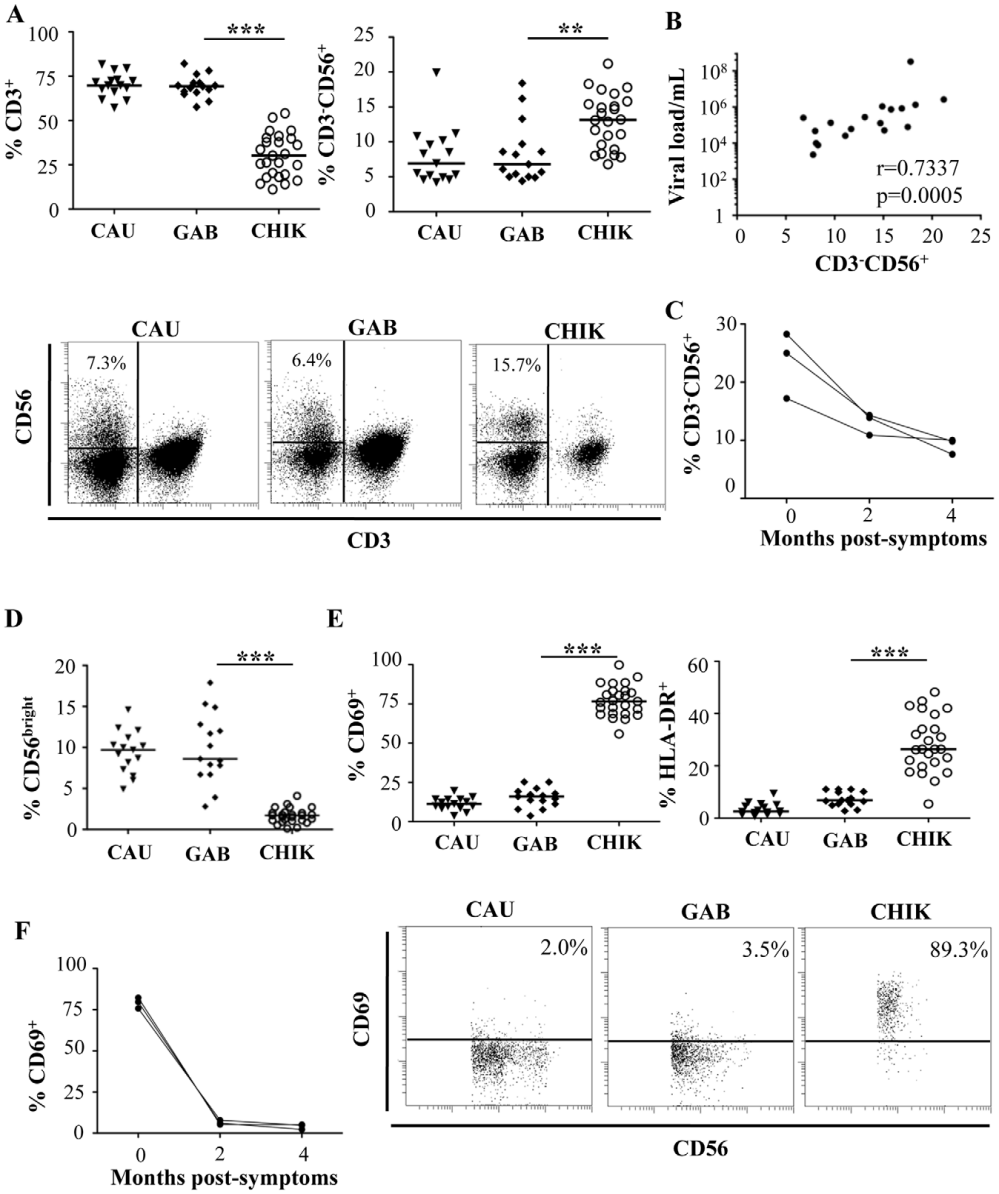
Distribution and activation status of NK cells from CHIKV-infected patients. (A) FACS analysis of percentages of CD3^+^ T cells and CD3^-^CD56^+^ NK cells from 15 Caucasian (CAU) and 15 Gabonese (GAB) healthy donors, and 25 CHIKV-infected patients (CHIK). Samples were gated on the lymphocyte gate for flow cytometric analysis. Horizontal bars represent the median values. Representative samples of each group are presented in the lower panels. Numbers are the percentage of CD3^-^CD56^+^ NK cells in the CD45^+^ lymphocyte gate. The region used to determine the proportion of CD3^-^CD56^+^ NK cells is shown. (B) Correlation between viral load and frequency of NK cells from CHIKV-infected patients. (C) Longitudinal studies of the percentage of CD3^-^CD56^+^ NK cells from 3 Caucasian CHIK-infected patients. (D) Frequency of CD56^bright^ cells among the CD3^-^CD56^+^ NK cells. (E) Expression of the activation markers CD69 or HLA-DR on NK cells. Samples were gated on the CD3^-^CD56^+^ NK-cell subset for flow cytometric analysis. Horizontal bars indicate the median. Representative samples of each group are presented in the lower panels. Numbers are the percentage of CD69^+^ cells in the CD3^-^CD56^+^ NK-cell gate. (F) Longitudinal study of the percentage of CD3^-^CD56^+^ NK cells expressing CD69, from 3 CHIKV-infected Caucasian patients. **: p<0.001; ***: p<0.0001.

Flow cytometry was used to assess the frequency of CD3^+^ T and CD3^-^CD56^+^ NK subsets in CHIKV-infected patients as well as in healthy Caucasian and Gabonese individuals. These lymphocyte subsets were found at similar frequencies in both healthy control groups, regardless of their origin. In contrast, and consistently with previous studies [14], [23], the percentage of CD3^+^ T cells was significantly lower in CHIKV-infected patients (p<0.0001) than in controls (Figure 1A). Infection-associated profound T lymphopenia tended to spare CD3^-^CD56^+^ NK cells, and the proportions of these cells increased significantly after CHIKV infection, to 13.0±3.9% compared with 8.4±4.3% in healthy Gabonese controls (p = 0.0006) (Figure 1A). The increase in NK-cell frequency was directly correlated with viral load (r = 0.7337; p = 0.0005) (Figure 1B). However, the absolute count of NK cells from the Caucasian CHIKV-infected patients (233±48 per mm^3^) was similar to that among the healthy Caucasian controls (275±66 per mm^3^). In contrast, the CD3^+^ T cell count was sharply lower in CHIKV-infected patients than in controls (Supplementary Table S1). These data are in line with a previous published study [23]. Of note, the modulation of frequencies of both CD3^+^ T and CD3^-^CD56^+^ NK cells was transient and rapidly returned to baseline levels after a two month-period (Figure 1C).

NK cells can be divided into CD56^bright^ and CD56^dim^ populations, based on the cell-surface density of the CD56 molecules; these subgroups present distinct phenotypic and functional properties [28]. Intriguingly, the percentage of CD56^bright^ NK cells was very significantly lower in CHIKV-infected patients than in healthy controls (p<0.0001) (Figure 1D), a finding that supports the hypothesis of profound upheavals of the NK cell subset after CHIKV infection.

The percentage of activated NK cells was also assessed by flow cytometric determination of the early activation marker CD69. As shown in Figure 1E, the NK cells from CHIKV-infected patients were activated; 77.2±9.9% of the NK cells expressed CD69 vs 15.4±6.2% in healthy donors (p<0.0001). Similar results were observed with HLA-DR, another activation marker (Figure 1E). Furthermore, within two months, the rate of NK cell activation reverted back to that of healthy donors (Figure 1F), in association with viral clearance (Supplementary Table S1).

### Major phenotypic features of NK cells from CHIKV-infected patients

**Figure 2:**
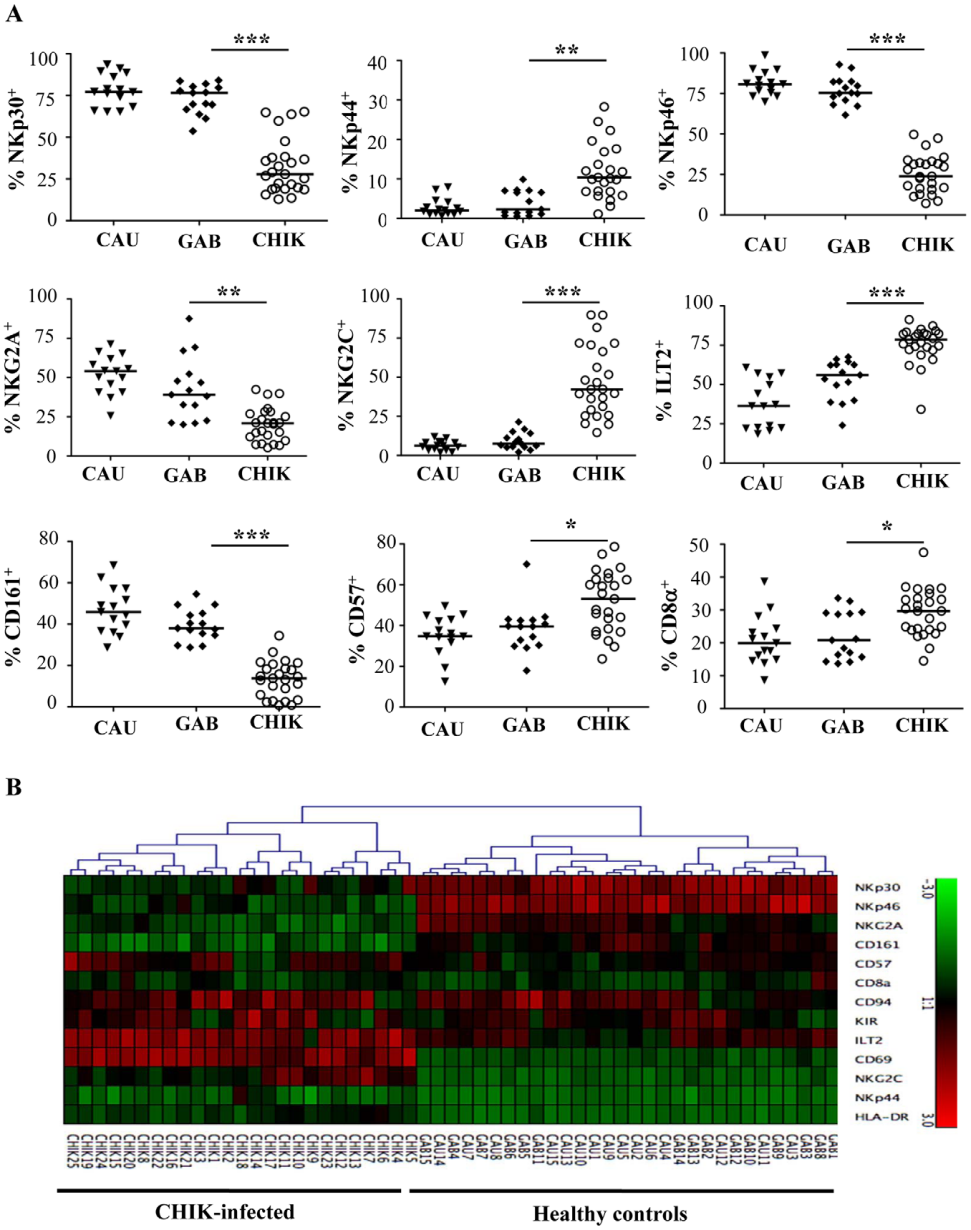
Patterns of specific NK receptors on CD3^-^CD56^+^ NK cells from CHIKV-infected patients. PBMC were collected from 15 Caucasian (CAU) and 15 Gabonese (GAB) healthy donors, and 25 CHIKV-infected patients (CHIK) to perform large-scale five-color flow cytometric analysis of CD3^-^CD56^+^ NK cells. (A) Frequency of the expression of NK-cell receptors including: NCR (NKp30, NKp44 and NKp46), NKG2A and NKG2C, ILT-2, as well as CD161, CD57, and CD8α. Horizontal bars indicate the median. *: p<0.05; **: p<0.001; ***: p<0.0001. (B) Hierarchical clustering of the 13 combinations of NK cell markers from healthy controls (Caucasian, CAU and Gabonese, GAB), and CHIKV-infected individuals (CHIK). Each horizontal line represents a particular NK-cell marker, and the color of each square reflects the percentage of cell-surface expression of the corresponding marker in each healthy or CHIKV-infected sample. Values measured for all samples were color displayed and rank ordered with the median for healthy donors considered the reference: green indicates below the median, and red indicates above the median, with values ranging from -3 and +3. Analysis was performed with the Genesis program (available at www.genome.tugraz.at).

To explore whether CHIKV infection was associated with the pattern of natural killer receptor (NKR) expression, flow cytometric analysis was performed with anti-CD3 and anti-CD56 mAbs, in combination with a panel of reagents for NKR. CHIKV-infected and healthy individuals did not differ significantly in their proportions of NK cells bearing NKG2D, 2B4, LAIR-1, and DNAM-1 (data not shown). In contrast, both the frequency of fluorescence intensity and mean fluorescence intensity (MFI) of NKp30 and NKp46, two specific natural cytotoxic receptors (NCR) constitutively expressed on virtually all NK cells, decreased significantly after CHIKV-infection (Figure 2A and Supplementary Figure S1). In contrast, NKp44, a marker usually expressed only upon activation, was upregulated in most infected patients, as were other CD69 and HLA-DR activation markers (Figures 1E and 2A). More importantly, the balance of inhibitory NKG2A to activating NKG2C cells switched during acute CHIKV infection. Thus, all infected patients showed a significant loss of both the frequency (p<0.0001) and MFI (p = 0.0005) of NKG2A^+^ NK cells, a loss corresponding to the dramatic expansion of NK cells expressing NKG2C activating receptors. The percentage of NKG2C^+^ cells increased after CHIKV infection, to 46.6±22.3% compared with 9.3±4.3% in healthy Gabonese controls (p<0.0001) (Figure 2A and Supplementary Figure S1A). Similarly, MFI of NKG2C increased significantly in CHIKV-infected patients, compared with controls (Supplementary Figure S1B). In addition, both the percentage and the MFI of NK cells expressing ILT-2, an inhibitory receptor that recognizes a broad range of classical MHC class-I molecules on surrounding cells, were higher in CHIKV-infected patients than controls. Specifically, around 77% of the NK cells from infected patients expressed ILT-2, compared with 53% of those from Gabonese and 38% from Caucasian healthy controls (Figure 2A and Supplementary Figure S1). This difference suggests an important expansion of a distinct population of activated NK cells that express NKG2C and ILT-2 but whose expression of NKG2A, NKp30 and NKp46 is skewed.

**Figure 3:**
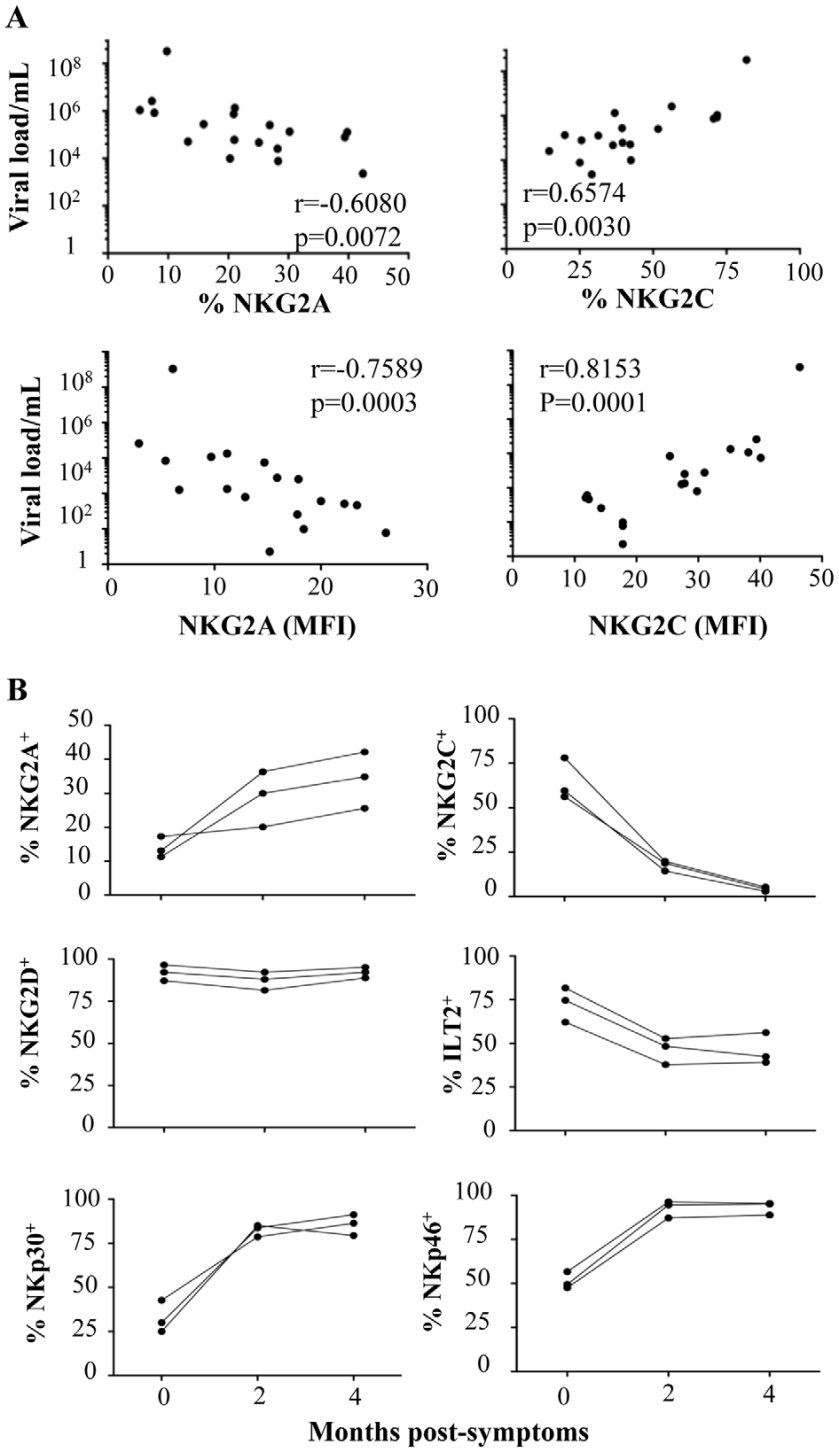
Correlation between viral load and NKR expression and kinetic expression of NKRs after CHIKV infection. (A) Correlation between viral load and NKG2A or NKG2C, both in frequency and MFI, on CD3^-^CD56^+^ NK cells from CHIKV-infected patients. NS: not significant. (B) Three Caucasian CHIKV-infected patients were tested at 0, 2, and 4 months after symptom onset, to determine the frequency of various NKRs, including NKG2A, NKG2C, NKG2D, ILT-2, NKp30, and NKp46, on CD3^-^CD56^+^ NK cells.

To address the specific characterization of the NKR repertoire from CHIKV-infected patients, hierarchical clustering analysis of CD3^-^CD56^+^ NK cells was performed. In an important finding, it showed that CHIKV-infected patients were easily distinguished from both sets of healthy controls and expressed homogeneous patterns of cell-surface markers (Figure 2B). This analysis did not distinguish the Caucasian from the Gabonese healthy controls. In addition, Spearman rank correlation analyses tested viral load versus NKR expression, which was modulated on NK cells from CHIKV-infected patients. They showed that only the expression of NKG2A and NKG2C (both their frequency and MFI) was significantly correlated with viral load (Figure 3A and Supplementary Figure S2). It is noteworthy that kinetic studies of the three viremic Caucasian CHIKV-infected patients, during the first four months post-symptoms, demonstrated that the NKR modulation was transient. Figure 3B shows that the overexpression of ILT-2 and NKG2C and the down-modulation of NKG2A, NKp30 and NKp46 were transient in all patients tested; the levels of all of these NKRs returned nearly to baseline within two months after symptoms stopped. Other markers, such as NKG2D, were not modulated after infection and remained consistent over time (Figure 3B). As noted above, the modulation of certain NKRs on NK cells was closely linked to the viral load, which ranged from 4.3×10^6^ to 6.0×10^7^ copies/ml at the time of symptom onset and became undetectable in all patients within two months (Supplementary Table S1).

### Preferential modulation of receptors for HLA-C1 alleles on NK cells from CHIKV-infected patients

**Figure 4:**
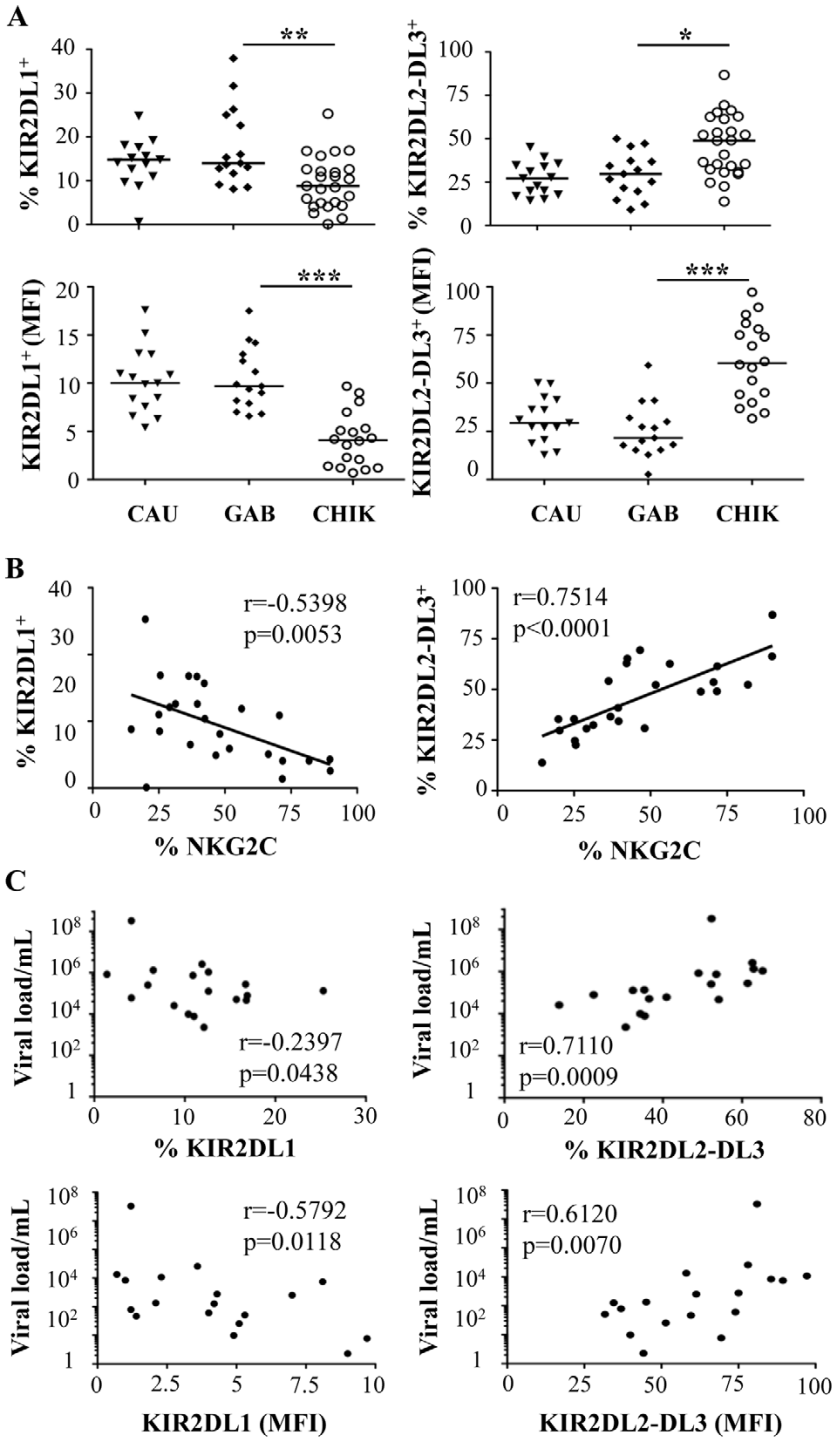
Specific modulation of HLA-Cw ligands on CD3^-^CD56^+^ NK cells from CHIKV-infected patients. PBMC were collected from 15 Caucasian (CAU) and 15 Gabonese (GAB) healthy donors, and 25 CHIKV-infected patients (CHIK). (A) Frequency and MFI of KIR2DL1 and KIR2DL2/DL3 on CD3^-^CD56^+^ NK cells. Horizontal bars indicate the median. *: p<0.05; **: p<0.001. (B) Correlation between expression of NKG2C and HLA-Cw ligands KIR2DL1 and KIR2DL2/DL3 on NK cells from the CHIKV-infected individuals. (C) Correlation between the viral load and KIR2DL1 or KIR2DL2/DL3 on CD3^-^CD56^+^ NK cells, both in frequency and MFI.

As previously shown, KIRs are stochastically expressed on the surface of NK cells and are critical for monitoring alterations in MHC expression during viral infection, as shown in the KIR gene cluster in the NCBI dbLRC database (http://www.ashi-hla.org/docs/pubs/abstracts/abs05/10.html). While acute CHIKV infection is associated with a high frequency of NK cells (Figure 1A), as described [23], it is uncertain whether this expansion occurs specifically in KIR-expressing NK cells. We therefore assessed the overall change in KIR^+^ NK cells with a pool of antibodies combining CD158a, CD158b, CD158e, and NKB1, and observed a similar frequency in both CHIKV-infected and healthy individuals (data not shown). However, when KIR markers were split during single KIR analysis, CHIKV-infected and control individuals had significantly different levels of receptors for HLA-C alleles. These included KIR2DL1, specific for HLA-C2 alleles, and KIR2DL2/DL3, specific for HLA-C1 alleles. Profiles for both frequency and MFI of inhibitory ligands for HLA-C molecules were similar (Figure 4A and Supplementary Figure S3A). Thus, compared with healthy controls, NK cells from CHIKV^+^ patients expressed significantly less KIR2DL1 (p = 0.0027) and significantly more KIR2DL2/DL3 (p = 0.0008) (Figure 4A). Thus, up to 76% of the NK cells in CHIKV-infected patients were stained with an anti-KIR2DL2/DL3 mAb. Importantly, KIR2DL1 expression in NK cells from CHIK-infected patients was significantly inversely correlated with their KIR2DL2/DL3 expression (p = 0.0318, r = −0.5201) (Supplementary Figure S3B). We therefore conducted several analyses to identify the underlying factors that might account for these differences observed in KIR2DL1 and KIR2DL2/DL3 expression during acute CHIKV infection. Intriguingly, we observed significant correlations between these receptors for HLA-C alleles and NKG2C: KIR2DL1 expression was inversely correlated with NKG2C expression (r = −0.5398, p = 0.0053), whereas KIR2DL2/DL3 expression was directly correlated with it (r = 0.7514, p<0.0001) (Figure 4B). These findings were strengthened by significant correlations between the viral load and expressions of both KIR2DL1 (r = −0.2397, p = 0.0438) and KIR2DL2/DL3 (r = 0.7110, p = 0.0009) (Figure 4C). Calculation of the MFI of inhibitory ligands for HLA-C molecules produced similar profiles (Figure 4C). Expression levels of other inhibitory KIRs (KIR1DL4, KIR2DL5, and KIR3DL1), and activating KIRs (KIR2DS1, KIR2DS2, KIR2DS4, and KIR3DS1) were similar in all samples, irrespective of viral load (Supplementary Figure S3C and data not shown). Importantly, in the nine CHIKV-infected Gabonese patients who had supplemental examinations, HLA-C genotyping revealed that 8 of them were HLA-C1/C1 or HLAC1/C2 (Table 1). Remarkably, the patients expressing the highest levels of KIR2DL2/DL3 on NK cells were those homozygous for HLA-CI/CI. Concomitantly, all of them expressed low levels of KIR2DL1, specifically recognized by HLA-C2 alleles, in relation to high viral load and NKG2C expression on NK cells (Table 1). It is important to note that Table 1 also shows that all Caucasian CHIKV-infected patients tested were HLA-C1/C1 or HLA-C1/C2, a genotype associated with high expression of NKG2C and KIR2DL2/DL3 and low expression of KIR2DL1.

**Table 1:** Association between viral load, expression of NKG2C and KIR2DL2/DL3, and HLA-C1 genotype in CHIKV-infected patients.

Samples (#)	Viral Load (copies/mL)	Phenotype (%)*			Genotype HLA-C	
		NKG2C	KIR2DL1	KIR2DL2/DL3	Alleles	Group
**Gabonese patients**						
1	7.8 E04	25.6	16.9	25.2	0701/0401	C2/C1
2	2.5 E04	14.6	11.5	13.8	1403/0702	C2/C2
3	1.3 E05	19.9	8.8	32.4	1601/0402	C2/C1
5	8.4 E05	71.7	1.4	49.0	1402/0304	C1/C2
6	7.7 E03	25.0	25.3	35.4	1201/1802	C2/C1
12	7.4 E05	70.6	10.9	53.5	0602/1703	C1/C1
13	2.6 E06	56.3	11.9	62.5	0602/0302	C1/C2
18	3.3 E08	81.8	4.1	52.3	0705/1701	C1/C1
20	1.1 E06	71.8	5.9	65.2	0401/0202	C1/C1
**Caucasian patients**						
1	2.3 E05	52.0	20.3	24.2	0101/0707	C1/C2
2	6.6 E06	55.7	14.8	37.3	0604/1301	C2/C1
3	5.1 E06	75.4	6.9	55.0	0302/0301	C1/C1

*Cell-surface expression on CD3^-^CD56^+^ NK cells

### Effective cytotoxicity of NK cells from CHIKV-infected patients

**Figure 5:**
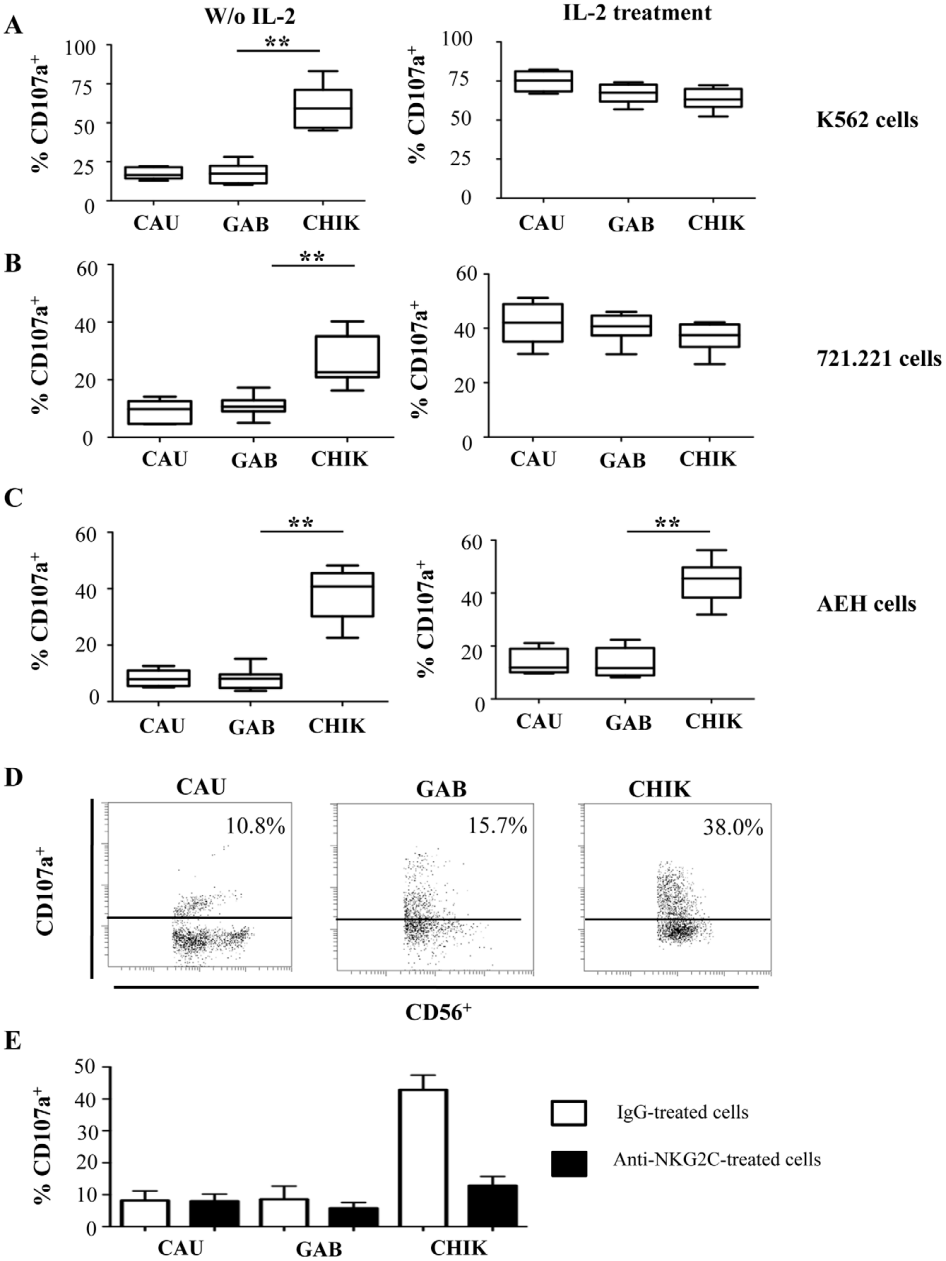
Robust degranulation efficacy of NK cells from CHIKV-infected patients against HLA-E^+^ target cells. PBMC were collected from 10 Caucasian (CAU) and 10 Gabonese (GAB) healthy donors, and 10 CHIKV-infected patients (CHIK) to determine the cytolytic capacities of NK cells, before and after a two-day treatment with IL-2. Degranulation activity was determined by CD107a expression against K562 (A), 721.221 (B), or HLA-E-transfected 721.221 (AEH-cells) (C) target cells. Results are shown for an effector/target (E/T) cell ratio of 1/1. Horizontal bars indicate the median. **: p<0.001. (D) Representative samples of each group were gated on the IL-2 activated CD3^-^CD56^+^ NK-cell subset in the presence of AEH HLA-E-expressing target cells. Numbers denote the percentage of CD107a^+^ cells among the CD3^-^CD56^+^ NK cells. (E) Neutralization of the degranulation in the presence of anti-NKG2C mAb. PBMC from 5 healthy Caucasian (CAU) and 5 healthy Gabonese (GAB) donors, and 5 CHIKV-infected patients (CHIK) were tested in the presence of neutralizing anti-NKG2C mAb (closed bars) or IgG isotypic control (Open bars) against HLA-E-transfected 721.221. Results are expressed in mean±SD.

To evaluate the functional capacities of NK cells from CHIKV-infected patients, non-activated and IL-2 activated PBMC were analyzed in a degranulation assay against K562, 721.221, and HLA-E-transfected 721.221 (AEH) target cells (Figure 5). In the absence of IL-2 activation, a minimal level of CD107a expression was detected at the surface of NK cells from healthy controls, regardless of the target cell line. In contrast, the degranulation capacity of NK cells from CHIKV-infected patients was strongly enhanced against HLA-class I negative K562 (p = 0.0005), and 721.221 (p = 0.0008) target cells (Figures 5A and 5B). More importantly, in the presence of HLA-E^+^ target cells, NK cells from CHIKV^+^ patients displayed greater cytotoxic activity than did NK cells from healthy donors (Figure 5C). These results are in accordance with the high expression level of major activation markers, including CD69, HLA-DR and NKp44, but also with NKG2C, the receptor of HLA-E. The intracellular production of perforin and granzyme-B did not differ between CHIKV-infected and healthy individuals (data not shown). Following IL-2 activation, CD107a expression on NK cells from CHIKV^+^ patients was similar to that in controls in the presence of K562 or 721.221 target cells. In contrast, in the presence of HLA-E^+^ target cells, degranulation capacity was enhanced in NK cells from CHIKV^+^ patients, compared with those from controls (p = 0.0414) (Figures 5C and 5D). To confirm the key role of NKG2C in the lytic capacity of NK cells from CHKIV^+^ samples, we repeated these experiments in the presence of neutralizing NKG2C mAb. The degranulation capacity of NK cells from five CHIKV^+^ patients decreased strongly in the presence of anti-NKG2C, to less than 12.4±4.6%, compared with 44.6±5.4% in the presence of an IgG isotypic control (Figure 5E).

### Downmodulation of intracellular IFN-γ expression in NK cells from CHIKV-infected patients

**Figure 6:**
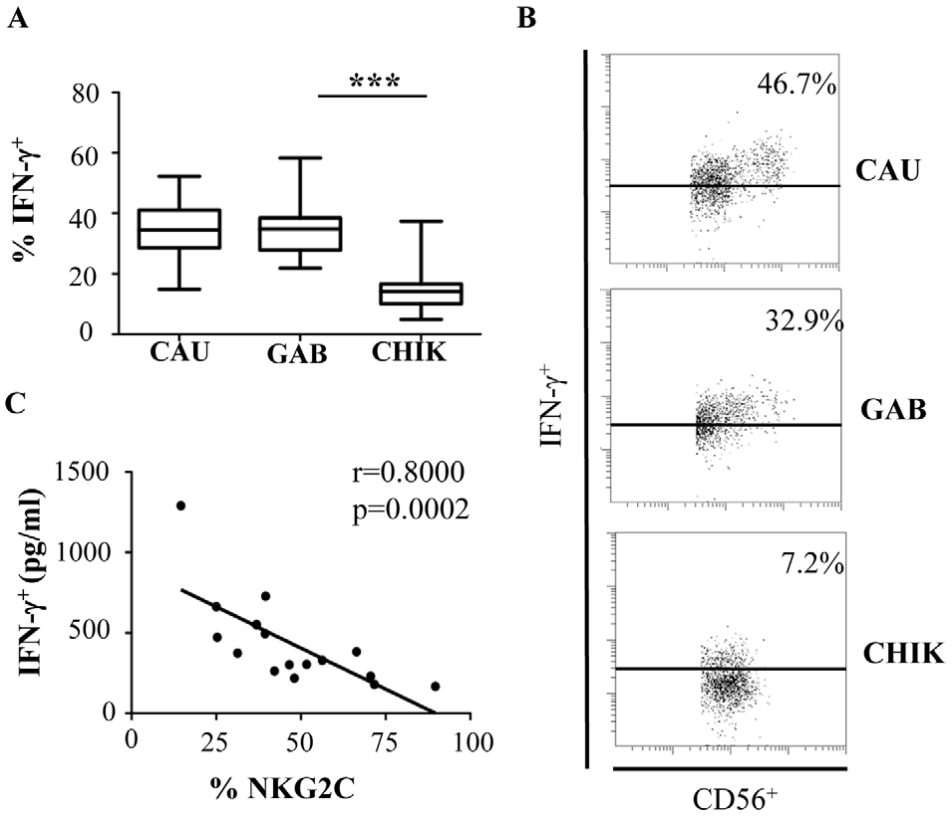
Significant down-modulation of intracellular IFN-γ expression in NK cells from CHIKV-infected patients. (A) PBMC were collected from 10 Caucasian (CAU), and 10 Gabonese (GAB) healthy donors, and 10 CHIKV-infected patients (CHIK) to determine intracellular production of IFN-γ after overnight treatment with IL-12 and IL-18. Horizontal bars indicate the median. ***: p<0.0001. (B) Representative samples of each group are shown. Samples were gated on the CD3^-^CD56^+^ NK-cell gate for flow cytometric analysis. Numbers denote the percentage of intracellular IFN-γ^+^ production among CD3^-^CD56^+^ NK cells. (C) Correlation between NKG2C expression by CD3^-^CD56^+^ NK cells and IFN-γ production in the sera of CHIKV-infected patients determined with Luminex Technology (Bio-Rad), as recently published [14].

In addition to their lytic activity, NK cells release several cytokines and chemokines that play a role in the recruitment and activation of the adaptive immune response [29]. The level of intracellular IFN-γ after treatment with IL-12 and IL-18 was significantly lower in CHIKV^+^ NK cells (p<0.0001) than among the healthy controls, regardless of their geographic origin or race (Figures 6A and 6B). These data are closely associated with the inverse correlation between the production of IFN-γ in the serum of CHIKV-infected patients and the level of NKG2C expression on NK cells (r = -0.8000; p = 0.0002) (Figure 6C).

In summary, these results provide strong support for the hypothesis that the expansion of NKG2C^+^ NK cells from CHIKV-infected individuals is associated with a dichotomy between cytolytic and immunoregulatory functions in NK cells during acute infection.

## Discussion

This paper describes an extensive phenotypic and functional study of NK cells from CHIKV-infected individuals and provides the first evidence that CHIKV infection may selectively shape the NKR repertoire of healthy individuals. NK cells in patients shortly after CHIKV infection had significantly lower levels of NKp30 and NKp46, a reduction undoubtedly linked to their high expression of activation markers (CD69 and HLA-DR), as previously described after HCMV and HIV-1 infections [30]–[32]. A hierarchical clustering analysis of a large panel of phenotypic markers of the NK cell subset strengthened the evidence of NKR repertoire modulation, showing that CHIKV-infected patients, have phenotypes quite distinct from those of either Caucasian or Gabonese healthy donors. This modulation likely reflects the challenge exerted by CHIKV on the innate immune system and thus may become a useful indicator for exploring the complex host-pathogen relation during the acute phase of infection.

In this study, we also demonstrated that CHIKV infection appears to impair the ability of NK cells to produce IFN-γ, as previously reported during such viral infections as HIV-1 and viral hepatitis [30], [33], [34], particularly at the peak of viremia. This impairment may be linked to the modulation in these patients of the subset of cells that produce IFN-γ most effectively — the CD56^bright^ NK-cell subset. Experimental evidence indicates that NK-cell development proceeds from a CD56^bright^ to CD56^dim^ phenotype [35]–[37]. Several other changes in NKR expression reinforce this concept that NK cells mature following CHIKV infection. For example, expression of CD57, which has been recognized as a marker of replicative senescence of T lymphocytes, increases markedly on NK cells shortly after CHIKV infection. This marker is known to be absent from cord blood NK cells and found at higher levels in the elderly than in young or middle-aged subjects [38]. Consistent with these data, CHIKV infection completely shifted the NK-cell repertoire to create a specific subset of highly mature CD56^dim^ NK cells mostly unresponsive to cytokine stimulation, but with strong cytolytic capacity for killing diseased host cells, according to their cell-activation phenotype. These data are in line with the recent study of Bjorkström et al [39] showing prompt NK cell activation and expansion in humans infected with hantavirus.

Our data show that NKG2C^+^ NK cells expand after CHIKC infection. However we cannot exclude an induction of NKG2C upon preexisting NK cells, or the elimination of NK cells that do not express NKG2C. This is closely associated with major modifications in the activation, differentiation, and cytolytic capacities of NK cells from CHIKV-infected viremic individuals. NKG2 receptor expression switched from inhibitory to activating after acute CHIKV infection, as previously described in other human infections, including HIV-1, CMV, HBV, and HCV [40]–[42]. The loss of NKG2A^+^ NK cells accompanied the expansion of NKG2C^+^ NK cells, which exist at very low frequencies in uninfected individuals. The frequency of NKG2C^+^ cells observed among CHIKV-infected individuals varied widely, but it is especially notable that it was significantly correlated with viral load. Interestingly, the rapid increased of NKG2C in response to the acute CHIKV infection was followed by a contraction phase, after viral clearance.

We cannot, however, reach a definitive conclusion about either the expansion of a specific NK cell subset or a difference in the lymphopenia rate in patients after CHIKV-infection, although both have been observed in a restricted population of murine NK cells expressing the activating receptor Ly49H^+^ after MCMV infection [43]–[45]. In this model, NK cells were shown to possess features previously attributed to cells of the adaptive immune system, including the expansion of a specific subset followed by contraction and formation of cells with memory-like characteristics [29], [45], [46]. NKG2C expression during acute hantavirus and HIV-1 infection has been associated with HCMV, but not EBV or HSV seropositivity [31], [39], [47]. Here we found that NKG2C expression was associated with HCMV serostatus in the three Caucasian CHIKV-infected patients. Evaluation of the HCMV status of the Gabonese patients was not relevant, as seroprevalence approaches 100% in this country [48].

More intriguingly, the NK cell repertoire appeared to be unusual with strong and specific correlations between NKG2C expression and specific self-inhibitory KIRs. In contrast to the overall NK cell repertoire, which contains a random distribution of KIRs, the NKG2C^+^ NK cells in CHIKV-infected patients are associated with HLA-Cw allele receptors. The correlation with KIR2DL1, recognizing C2 HLA-C subtypes, is inverse, while that with KIR2DL2/DL3, recognizing C1 subtypes, is direct. This suggests that NK cells coexpressing NKG2C and receptors for HLA-C1 alleles expand during an acute viral infection. More importantly, HLA-C genotyping revealed that 11/12 CHIKV-infected patients examined in detail had HLA-C1/C1 or HLA-C1/C2 alleles. This finding suggests that expansion of highly cytotoxic NKG2C^+^ NK cells is associated with imbalanced expression of unique self-specific receptors and may dampen autoreactivity and limit immunopathology in CHIKV-infected patients.

Analysis of a large number of CHIKV-infected individuals, combining genetic analysis of KIR and HLA, kinetic study of phenotypic and functional features of NK cells, and the long-term evolution of clinical indicators, might make it possible to draw statistically powerful conclusions. The presence of a single inhibitory HLA-C binding KIR showed that the KIR overexpressed during CHIKV-infection mediates NK-cell licensing in the highly functional NKG2C^+^ NK cell subset, protecting against autoreactivity. This finding is supported by our recent observation of a persistent indolent proliferation of CD3^-^CD56^+^ large granular lymphocytes expressing NKG2C^+^ [49]. These data are also consistent with a case report of acute HCMV infection in a 3-month-old girl whose genes encoded a deficient alpha-chain of the IL-7 receptor known to be associated with a SCID-phenotype; phenotype characterization showed an extreme transient amplification of NKG2C-bearing NK cells positive for KIR2DL2/DL3, which accounted for more than 80% of the leukocytes [50]. More recently, Björkström *et a*l [39] also showed a clonal selection of educated CD56^dim^NKG2C^+^KIR^+^ NK cells in 5 hantavirus-infected patients. Taken together, these important data suggest a certain level of clonality in the response of NK cells against acute infection, including CHIKV infection. However, the underlying mechanism mediating this effect remains to be defined.

Collectively, our data suggest that the clonal expansion of a unique subset of NK cells coexpressing NKG2C and receptors for HLA-C1 alleles and correlated with the viral load, suggests that NK cells are able to sense CHIKV from the beginning of infection and may thus contribute to viral clearance. Understanding how these complex innate responses affect the outcome of CHIKV infection will help in the development of vaccines or other therapeutic strategies that could use innate immunity to enhance viral control with minimal pathogenesis.

## Materials and Methods

### Human ethics statement

This study was conducted in accordance with the principles expressed in the Declaration of Helsinki and with French statutory and regulatory law. Patients received information about research performed on biological samples and provided written informed consent to participate. The hospital's institutional review board (Comité de protection des personnes Ile-de-France VI) approved the study (Hôpital Pitié-Salpêtrière, 47 boulevard de l'Hôpital, 75013 Paris).

### Patients and healthy controls

Peripheral blood samples from 25 CHIKV-infected patients (mean age 32±15 years, 60% women) were obtained during the CHIKV outbreak that occurred in Gabon, Central Africa, between March and August 2007. The outbreak began first in Libreville, the capital, and then spread to several small towns located on the road towards Cameroon in northern Gabon, generating approximately 20,000 cases. These samples were collected from patients whose symptoms included fever, arthralgia, and asthenia and who visited specific medical centers in Libreville during the first five days after the onset of symptoms. Diagnosis of CHIKV infection was confirmed for each of these patients, as described [7], [14]. These CHIKV-infected patients were negative for dengue fever, yellow fever, West Nile fever, Rift Valley fever, and malaria. Gabonese (n = 15) and Caucasian (n = 15) sex- and age-matched healthy volunteers from Franceville (Gabon) and our hospital blood bank (EFS, Pitié-Salpêtrière Hospital, Paris, France) were used as controls. Kinetic studies were performed on 3 Caucasian CHIKV-infected patients from the Department of Infectious Diseases of the Pitié-Salpêtrière Hospital (Paris, France) in 2006.

### Viral RNA extraction and quantification

Viral RNA was extracted from 90 µl of plasma from CHIKV-infected patients with an EZ1 advanced XL system (Qiagen, Courtaboeuf, France) and the EZ1 virus kit V2.0 (Qiagen). With the high capacity cDNA reverse transcriptase kit (Applied Biosystems, Foster City, CA, USA) and according to the manufacturer's recommendations, 25 µl of extracted RNA was immediately reverse transcribed into cDNA. Real-time PCR was carried out in a 7500 real-time PCR system (Applied Biosystems), with the Universal PCR master mix kit (Applied Biosystems) according to the manufacturer's recommendations with the specific primers F-CHIK and R-CHIK and the probe P-CHIK (10 mM) designed by Pastorino *et al*. [51]. A quantified synthetic CHIKV RNA transcript, kindly provided by the University of the Méditerranée (Pr X. De Lamballerie, Marseilles, France), was used as a standard. All amplifications were performed in duplicate.

### Flow cytometric analysis

NK cells were analyzed after staining with an appropriate antibody cocktail: anti-CD45 (J33), anti-CD3-ECD (UCHT1), anti-CD56-PC7 (N901), anti-CD8a-APC (B9.11), anti-CD159a/NKG2A-APC (Z199), anti-CD336/NKp44-PE (Z231), anti-CD335/NKp46-PE (BAB281), anti-NKG2D-PE (ON72), anti-CD85j/ILT2-PE (HP-F1), anti-CD69-APC (FN50), and anti-HLA-DR-PE (Immu357), KIR2DL1/KIR2DS1-PE (EB6B), anti-KIR2DL2/KIR2DL3/KIR2DS2-PE (GL183), KIR3DL1/KIR3DS1-PE (Z27) from Beckman Coulter; anti-CD94-FITC (HP-3D9), anti-CD57-FITC (S-HCL-1), and anti-CD161-FITC (DX12) from Becton Dickinson; anti-NKG2C-PE (134591), KIR2DS4-PE (179315), and anti-KIR2DL4-APC (181703) from R&D systems; anti-CD337/NKp30-PE (AF29-4D12), anti-KIR2DL1-APC (11PB6), anti-KIR2DL2/KIR2DL3-APC (DX27), and anti-KIR3DL1-APC (DX9) from Miltenyi Biotech; and anti-KIR2DL5-Alexa Fluor 647 (UP-R1) from e-biosciences. For intracellular staining, PBMC were fixed and permeabilized with a cytofix/cytoperm kit (Becton Dickinson) and stained with perforin-PE (δG9), or granzyme-B-FITC (GB1), as described [36]. Isotype-matched immunoglobulins served as negative controls. Lymphocytes were identified by characteristic forward and side scatter parameters and CD45 expression. Populations of interest were gated on patterns of CD56/CD3 staining within the CD45^+^ lymphocyte population. Results are expressed as the percentage of positive cells within the gated population. At least 20,000 CD45^+^ cells were analyzed on a Navios cytometer (Beckman Coulter). Expression of each NKR was measured as a percentage of the total CD3^-^CD56^+^ NK cells. Hierarchical clustering was applied, and the results were displayed with the use of the Genesis program (software available at www.genome.tugraz.at), as previously described [52]–[54].

### Functional analysis

Degranulation was assessed by the detection of LAMP1/CD107a, on PBMC cultured in the absence or in the presence of 1000 IU/ml of Proleukin-2 (Chiron) for 48 h, against K562, 721.211 or HLA-E-transfected 721.221 (AEH) target cell lines. Briefly, PBMC were resuspended in the presence of anti-CD107a mAb (H4A3, Becton Dickinson) with target cells at an effector:target (E:T) cell ratio of 1∶1. After 1 h of incubation, monensin (Sigma Aldrich) was added at 2 mM for an additional 4 h of incubation [36], [38].

To stimulate IFN-γ production, PBMC were incubated overnight in the presence of IL-12 (10 ng/ml) and IL-18 (100 ng/ml) (R&D Systems). Cells were then fixed and permeabilized with a cytofix/cytoperm kit (Becton Dickinson) and stained with anti-IFN-γ mAb (B27; Becton Dickinson), as described [36].

### HLA-Cw typing

Genomic DNA was extracted from PBMC of CHIKV-infected patients with the QIAmp DNA mini kit (Qiagen). HLA-C alleles were sequenced with the SBT kit (Aria Genetics). Sequences were read with a 3100 Genetic analyzer (Applied Biosystems) and computer-assisted Conexio genomics software.

### Statistical analysis

All statistical analyses were performed with Prism-5 software (GraphPad Software). Intergroup comparisons were assessed with the non-parametric Kruskal-Wallis test, with Dunn's post test to define the significance between results from 3 independent groups of subjects. Significance was defined by *P* less than 0.05 with a 2-tailed test. *p<0.05, **p<0.01, ***p<0.001**.** Nonparametric correlations were assessed by determination of the Spearman's rank correlation coefficient.

## Supporting Information

Figure S1 **Cell-surface expression of NK cell markers on NK cells from Caucasian (CAU) and Gabonese (GAB) healthy donors, and CHIKV-infected patients (CHIK).** (A) PBMC from one representative sample of each group of samples were stained with specific antibodies against NKG2A, NKG2C, ILT-2, NKp30 and NKp46, and then gated on the CD3^-^CD56^+^ NK-cell gate for flow cytometric analysis. Numbers denote the percentage of positive cells in the CD3^-^CD56^+^ NK-cell gate. (B) MFI of the expression of NK-cell receptors including NKG2A, NKG2C, ILT-2, NKp30 and NKp46. Horizontal bars indicate the median. *: p<0.05; **: p<0.001; ***: p<0.0001.
(TIF)

Figure S2 **Expression of KIR in CHIKV-infected patients.** (A) Expression of KIR2DL1 and KIR2DL2/DL3 on NK cells from one representative sample of each group, including Caucasian (CAU) and Gabonese (GAB) healthy donors, and CHIKV-infected patient (CHIK). Numbers denote the percentage of positive cells in the CD3^-^CD56^+^ NK-cell gate. (B) Correlation between KIR2DL1 and KIR2DL2/DL3 expression on CD3^-^CD56^+^ NK cells from CHIKV-infected patients. (C) Frequency and MFI values of KIR2DL4, KIR2DL5, and KIR3DL1 on NK cells from Caucasian (CAU), and Gabonese (GAB) healthy donors, and CHIKV-infected patients (CHIK).
(TIF)

Figure S3 **Expression of KIR in CHIKV-infected patients.** (A) Expression of KIR2DL1 and KIR2DL2/DL3 on NK cells from one representative sample of each group, including Caucasian (CAU) and Gabonese (GAB) healthy donors, and CHIKV-infected patient (CHIK). Numbers denote the percentage of positive cells in the CD3^-^CD56^+^ NK-cell gate. (B) Correlation between KIR2DL1 and KIR2DL2/DL3 expression on CD3^-^CD56^+^ NK cells from CHIKV-infected patients. (C) Frequency and MFI values of KIR2DL4, KIR2DL5, and KIR3DL1 on NK cells from Caucasian (CAU), and Gabonese (GAB) healthy donors, and CHIKV-infected patients (CHIK).
(TIF)

Table S1 **Viral load, absolute count and frequency of CD3^+^ T and CD3^-^CD56^+^ NK cells from Caucasian CHIKV-infected patients.**
(DOC)
