# Supplementary figures and images for: Unconventional Repertoire Profile Is Imprinted during Acute Chikungunya Infection for Natural Killer Cells Polarization toward Cytotoxicity
Source: PLoS Pathog. 2011 Sep 22;7(9):e1002268. doi: 10.1371/journal.ppat.1002268 (PMC3178577; doi:10.1371/journal.ppat.1002268)

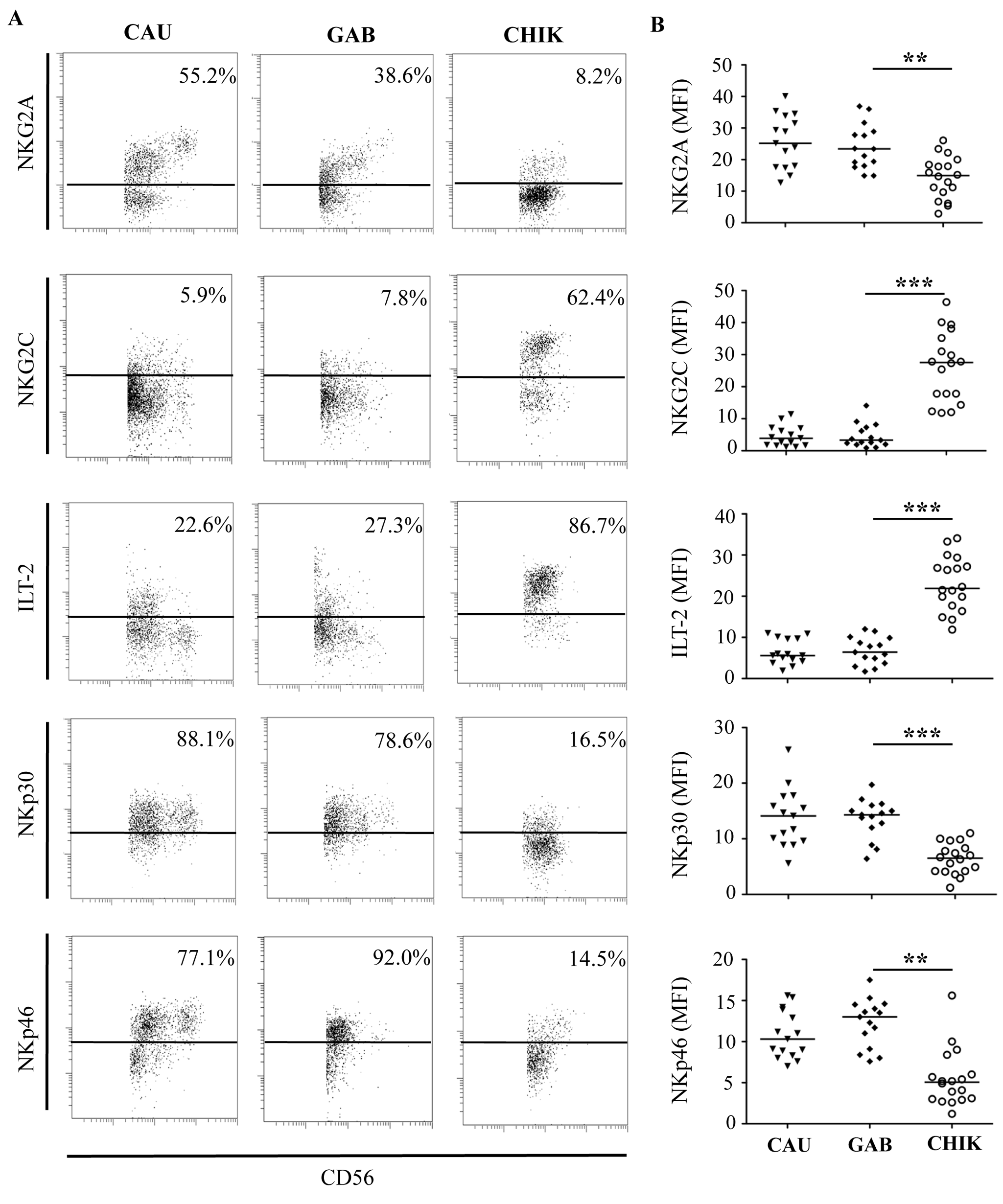

Supplement: Figure S1 — Cell-surface expression of NK cell markers on NK cells from Caucasian (CAU) and Gabonese (GAB) healthy donors, and CHIKV-infected patients (CHIK). (A) PBMC from one representative sample of each group of samples were stained with specific antibodies against NKG2A, NKG2C, ILT-2, NKp30 and NKp46, and then gated on the CD3-CD56+ NK-cell gate for flow cytometric analysis. Numbers denote the percentage of positive cells in the CD3-CD56+ NK-cell gate. (B) MFI of the expression of NK-cell receptors including NKG2A, NKG2C, ILT-2, NKp30 and NKp46. Horizontal bars indicate the median. *: p<0.05; **: p<0.001; ***: p<0.0001. (TIF) [file ppat.1002268.s001.tif]

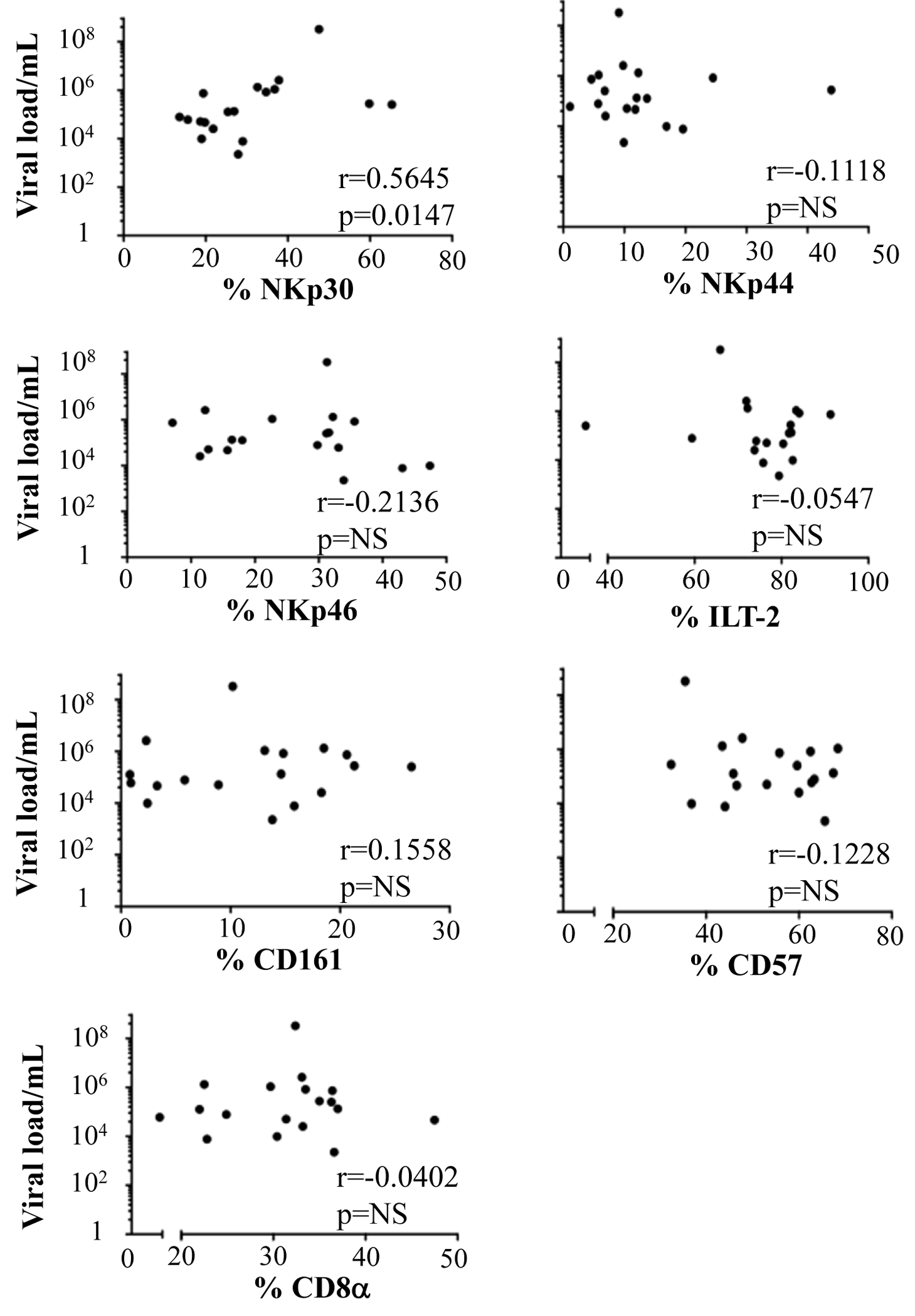

Supplement: Figure S2 — Expression of KIR in CHIKV-infected patients. (A) Expression of KIR2DL1 and KIR2DL2/DL3 on NK cells from one representative sample of each group, including Caucasian (CAU) and Gabonese (GAB) healthy donors, and CHIKV-infected patient (CHIK). Numbers denote the percentage of positive cells in the CD3-CD56+ NK-cell gate. (B) Correlation between KIR2DL1 and KIR2DL2/DL3 expression on CD3-CD56+ NK cells from CHIKV-infected patients. (C) Frequency and MFI values of KIR2DL4, KIR2DL5, and KIR3DL1 on NK cells from Caucasian (CAU), and Gabonese (GAB) healthy donors, and CHIKV-infected patients (CHIK). (TIF) [file ppat.1002268.s002.tif]

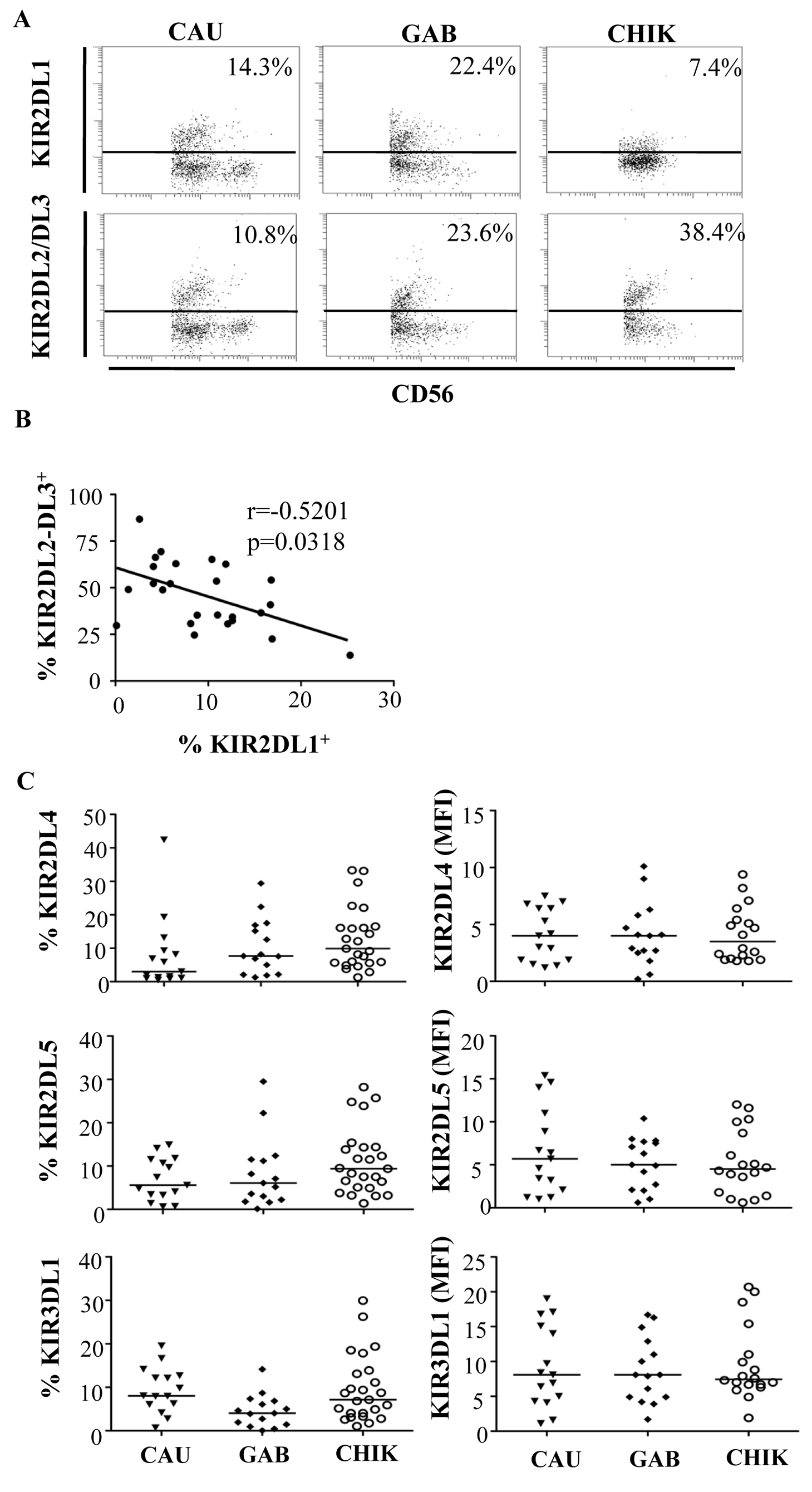

Supplement: Figure S3 — Expression of KIR in CHIKV-infected patients. (A) Expression of KIR2DL1 and KIR2DL2/DL3 on NK cells from one representative sample of each group, including Caucasian (CAU) and Gabonese (GAB) healthy donors, and CHIKV-infected patient (CHIK). Numbers denote the percentage of positive cells in the CD3-CD56+ NK-cell gate. (B) Correlation between KIR2DL1 and KIR2DL2/DL3 expression on CD3-CD56+ NK cells from CHIKV-infected patients. (C) Frequency and MFI values of KIR2DL4, KIR2DL5, and KIR3DL1 on NK cells from Caucasian (CAU), and Gabonese (GAB) healthy donors, and CHIKV-infected patients (CHIK). (TIF) [file ppat.1002268.s003.tif]
