# Supplementary material for: Unconventional Repertoire Profile Is Imprinted during Acute Chikungunya Infection for Natural Killer Cells Polarization toward Cytotoxicity
Source: PLoS Pathog. 2011 Sep 22;7(9):e1002268. doi: 10.1371/journal.ppat.1002268 (PMC3178577; doi:10.1371/journal.ppat.1002268)
Supplement: Table S1 — Viral load, absolute count and frequency of CD3+ T and CD3-CD56+ NK cells from Caucasian CHIKV-infected patients. (DOC) [file ppat.1002268.s004.doc]

**Table S1: Viral load, absolute count and frequency of CD3^+^ T and CD3^-^CD56^+^** NK cells from Caucasian CHIKV-infected patients.

| Patients (#) | Visit^a^ | Viral Load  (copies/ml) | CD3^+^ cells | | | CD3^-^CD56^+^ cells | | | NKG2C/NK cells^b^ | |
| --- | --- | --- | --- | --- | --- | --- | --- | --- | --- | --- |
|  |  |  | mm^3^ | % | | mm^3^ | % | | mm^3^ | % |
| 1 | T0 | 2.3 E05 | 684 | 57 | | 216 | 18 | | 119 | 55 |
|  | M2 | Negative | 1419 | 70 | | 223 | 11 | | 24 | 11 |
|  | M4 | Negative | 1518 | 72 | | 232 | 11 | | 2 | 1 |
| 2 | T0 | 6.6 E06 | 290 | 40 | | 196 | 27 | | 104 | 53 |
|  | M2 | Negative | 1316 | 69 | | 248 | 13 | | 27 | 11 |
|  | M4 | Negative | 1618 | 77 | | 231 | 11 | | 5 | 2 |
| 3 | T0 | 5.1 E06 | 358 | 36 | | 289 | 29 | | 217 | 75 |
|  | M2 | Negative | 1421 | 62 | | 252 | 11 | | 30 | 12 |
|  | M4 | Negative | 1542 | 70 | | 222 | 8 | | 4 | 2 |
| Ctl^c^ |  | | | | | | | | | |
| Mean | | NA | 1427 | | 75 | 285 | | 12 | 15 | 5 |
| SD | | NA | 300 | | 5 | 66 | | 4 | 12 | 4 |

^a^ Visit correspond to sampling at the first visit (T0), and at two (M2), and four (M4) months post-symptoms.

^b^ NKG2C^+^/NK cells correspond to the absolute count or frequency of NK cells expressing NKG2C.

^c^ Ctl correspond to the 15 Caucasian healthy controls. The results are expressed in mean and standard deviation (SD).

NA: Not Applicable
